# Genotypic and phenotypic characterization of biofilm production by *Staphylococcus aureus* strains isolated from bovine intramammary infections in Colombian dairy farms

**DOI:** 10.1016/j.heliyon.2019.e02535

**Published:** 2019-10-14

**Authors:** G. Torres, K. Vargas, M. Sánchez-Jiménez, J. Reyes-Velez, M. Olivera-Angel

**Affiliations:** a Tropical Medicine Colombian Institute, CES University, Cra. 43A No. 52 sur-99 Sabaneta, Antioquia, Colombia; b Biogenesis Research Group, Faculty of Agricultural Sciences, University of Antioquia, Cra 75 No. 65-87, Medellín, Antioquia, Colombia

**Keywords:** Microbiology, Animal science, Bacteria, Microbial genomics, Microorganism, Biofilms, Genotype, Mastitis, *Staphylococcus aureus*

## Abstract

The ability of *Staphylococcus aureus* to form biofilms is an important virulence factor because this has been associated with persistent bovine intramammary infections. Different mechanisms of biofilm formation have been described in *S. aureus*; however, the process has been found to be mainly driven by the *ica* and *bap* genes. The presence of the *ica* and *bap* genes, as well as the biofilm formation *in vitro* were evaluated in 229 *S. aureus* strains isolated from bovine milk collected from different regions of Department of Antioquia, Colombia. Three different genotypes grouped into three separate clusters were identified from *in vitro* assays. Genotype 1 (*ica* positive and *bap* negative) was the most prevalent (78.17%), followed by genotype 2 (*ica* and *bap* positive) (12.66%) and genotype 0 (*ica* and *bap* negative) (9.17%). Biofilm formation was observed in 81.26% of the strains from which 100% of genotype 2 isolates showed biofilm formation. The biofilms formed by genotype 2 isolates were also found to have the highest optical density (>2.4). These results showed that most of the *S. aureus* strains were capable of biofilm formation, suggesting the virulence potential particularly in *bap*-positive strains.

## 1 Introduction

*S. aureus* is one of the most common pathogens causing bovine intramammary infections (IMI), which are characterized by the presence of persistent microorganisms along with a poor response to antibiotic therapy. Animals with chronic infections act as important pathogen reservoirs, maintaining the occurrence of *S. aureus* infections in herds, which leads to significant economic losses in dairy farms (Schukken et al., 2011; Veh et al., 2015).

The ability of *S. aureus* to persist in the mammary gland has been associated with multiple virulence factors. Among these, biofilm formation is one of the most important because biofilms confer protection against antibiotics and the host immune response, which are key elements in the elimination of infections (Gomes et al., 2016). Previous research has shown that the biofilm formation process is both diverse and redundant (Zapotoczna et al., 2016). The two most studied mechanisms responsible for biofilm formation are polysaccharide intercellular adhesion (PIA)-dependent biofilms, associated with *ica* operon, and PIA-independent biofilms, generally mediated by biofilm-associated protein (Bap), is encoded by the *bap* gene (McCarthy et al., 2015).

Most clinical isolates that cause infections in both in humans and animals carry the *ica* locus; whereas the *bap* gene has only been found in strains isolated from cattle (Cucarella et al., 2004; Vautor et al., 2008). The *bap* gene in *S. aureus* is contained in a transposon that is part of SaPIbov2, a mobile pathogenicity island (Cucarella et al., 2004). Some reports have indicated that *bap*-positive strains generally are strong biofilm formers, even in the absence of the *ica* locus, and can cause more persistent infections than *bap*-negatives (Cucarella et al., 2004; Lasa and Penadés, 2006).

Several studies in different countries have evaluated the capacity to produce biofilms of *S. aureus* isolated from cattle (Cucarella et al., 2004; Serray et al., 2016; Marques et al., 2017; Notcovich et al., 2018). In Colombia however, where this pathogen is one of the major causes of IMI in dairy cows (Vidal et al., 2016), biofilm formation ability remains unknown. The aims of this study were to determine the distribution of the biofilm formation genes in *S. aureus* strains isolated from bovine IMI, as well as to evaluate their ability to form biofilms under *in vitro* conditions.

## 2 Materials and methods

### 2.1 Bacterial isolates

A total of 229 *S. aureus* strains recovered from bovine IMI were characterized in this study. Strains were isolated between July to December 2015 at the Laboratory of Microbiology (School Veterinary Medicine, Antioquia University, Medellín, Colombia), using the standard protocol recommended by National Mastitis Council (National Mastitis Council, 2004). Infected cows belonged to 91 dairy farms located in five regions (15 municipalities) from the Department of Antioquia, Colombia (Table 1). Strains were stored at −80 °C in Trypticase soy broth (TSB) (Oxoid, United Kingdom) supplemented with 10% glycerol until use.

**Table 1:** *S. aureus* strains isolated from cattle from each region.

Region	No. Municipalities	No. Isolates
North	7	164
Metropolitan area	1	26
East	5	21
South-west	1	17
West	1	1
Total	**15**	**229**

### 2.2 DNA extraction

*S. aureus* strains stocks were thawed and cultured on Trypticase soy agar (TSA) (Oxoid, United Kingdom). Plates were incubated at 37 °C for 24 h under aerobic conditions. DNA was extracted using DNeasy Blood & Tissue kit (Qiagen, Germany) according to protocol for Gram-positive bacteria. Amount and purity of DNA were measured using NanoDrop (ThermoFisher Scientific, USA). Extracted DNA was stored at −20 °C until use.

### 2.3 PCR assays

Phenotypic identification of strains was confirmed by Polymerase Chain Reaction (PCR) amplification of the *nuc* gene, which encodes for a thermostable nuclease specific for this pathogen (Brakstad et al., 1992). PCR was carried out as described by Fournier et al. (2008). The expected amplified fragment was 664 bp.

PCR to detect the biofilm-associated *ica* and *bap* genes was performed using primers and conditions previously described by Cucarella et al. (2004). The size of amplicons expected for *ica* and *bap* were 616 bp and 971 bp, respectively*.*

Each PCR mixture had a final volume of 25 μL, consisting of 1X Platinum PCR SuperMix (Invitrogen, USA), 0.2 μM of each primer, and 100 ng of DNA template. Reactions were performed in a C1000 Thermal Cycler (Bio-Rad, USA). Amplified products were visualized by electrophoresis on 1% agarose gels stained with ethidium bromide. Experiments were performed in triplicate. *S. aureus* strain V329 (provided by Dr. José Penades, University of Glasgow) was used as a positive control, which harbors both the analyzed genes.

### 2.4 Biofilm production assay in vitro

The biofilm formation ability of the different *S. aureus* strains was evaluated according to published protocols by Stepanović et al. (2007). The strains were transferred from stock culture into TSA and incubated at 37 °C overnight under aerobic conditions. The next day, colonies were suspended in sterile distilled water until a turbidity comparable to 0.5 MacFarland scale (∼10^8^ CFU/mL) was reached. This suspension was diluted 1:100 in TSB supplemented with 1% glucose (Merck, USA) to reach a bacterial concentration of approximately 10^6^ CFU/mL. Then, 200 μL from the diluted suspension was aliquoted into 96-well polystyrene tissue culture-treated microtiter plate (NEST, China) and incubated at 37 °C for 24 h under static aerobic conditions. The next day the wells were aspirated, and each well was washed three times with 300 μL sterile phosphate-buffered saline (PBS, pH 7.2). After washing, the remaining attached bacteria were fixed with 150 μL of methanol, and incubated at room temperature for 20 min. The methanol was discarded and plates were left inverted overnight to dry. Finally, the biofilm formed was stained with 150 μL of 2% crystal violet for 15 min. Plates were washed, and dye bound to the cells was eluted with 95% ethanol. The absorbance was measured at 570 nm using a microplate reader (Bio-Rad, USA). Experiments were performed in duplicate and repeated three times. The cut-off value (OD_C_) was defined as three standard deviations above the mean OD of the negative control (NC). The strains were classified within of the following categories: non-biofilm former (OD_570_ ≤ OD_C_) and biofilm former (OD_570_ > 2X OD_C_). *S. aureus* strains V329 (*ica* and *bap* positive) and ATCC 6538 (*ica* positive and *bap* negative) were used as positive controls, whereas TSB with glucose was used as a NC.

### 2.5 Hierarchical clustering analysis

The cluster analysis was performed using the R package “cluster” daisy function (version 2.0.8), which computes all the pairwise dissimilarities distances between isolates. This approach allows mixed types of variables (nominal, ordinal, asymmetric binary). This took into account both genotypes and the OD from each strain. The number of clusters was determined through a hierarchical clustering algorithm using the newly formed distance matrix.

### 2.6 Statistical analysis

A descriptive analysis was performed for the distribution of the genes of interest as well as biofilm production measured by the OD. Moreover, non-parametric tests were calculated using Kruskall–Wallis and unpaired Wilcoxon test for multiple comparisons between each genotype and clusters, with a statistical significance of *P* < 0.05. A combined variable between the presence of *ica* and *bap* genes was created, in order to determine the OD pairwise differences between clusters and the combined genotype variable. All analyses were carried out with R statistical software (version 3.6.0) (https://cran.R-project.org).

## 3 Results

### 3.1 Detection of *nuc* gene and biofilm-related genes

The *nuc* gene was present in all isolates, confirming that the strains evaluated were *S. aureus*. The PCR analysis identified the *ica* locus in 208 (90.82%) strains, and 29 (12.66%) of these also harbored the *bap* gene. Moreover, 21 (9.17%) strains did not amplify any of the biofilm markers (Table 2).

**Table 2:** Genotypes identified in *S. aureus* isolates.

Genotype	Markers	No. Of strains (%)
1	*ica*(+)–*bap*(-)	179 (78.17)
2	*ica*(+)–*bap*(+)	29 (12.66)
0	*ica*(-)–*bap*(-)	21 (9.17)
Total		**229 (100)**

(+) amplified marker; (-) marker not amplified.

### 3.2 Assessment of biofilm formation in vitro

The OD measured on microplates showed a wide distribution of values, ranging from a minimum of 0.10 to a maximum of 3.90 (mean = 1.19, median = 0.70). The mean OD value of NC (OD_NC_) was 0.12. *S. aureus* strains with OD values higher than three times the standard deviation of the mean OD_NC_ (OD > 0.23) were considered biofilm formers. According to this criterion, a total of 186 (81.26%) strains were biofilm producers. All the isolates (29/29–100%) from genotype 2 were biofilm formers; whereas 21.23% (38/179) and 23.81% (5/21) of the isolates from genotypes 1 and 0, respectively, were classified as non-biofilm formers (Table 3).

**Table 3:** Distribution of the biofilm-forming and non-biofilm-forming *S. aureus* strains by genotype.

Genotype	No. of strains (%)		Total
	Non-biofilm former	Biofilm former	
1	38 (21.23)	141 (78.77)	179 (78.17)
2	0	29 (100)	29 (12.66)
0	5 (23.81)	16 (76.19)	21 (9.17)
Total	**43 (18.74)**	**186 (81.26)**	229

### 3.3 Clusters obtained by hierarchical clustering algorithm

**Fig. 1:**
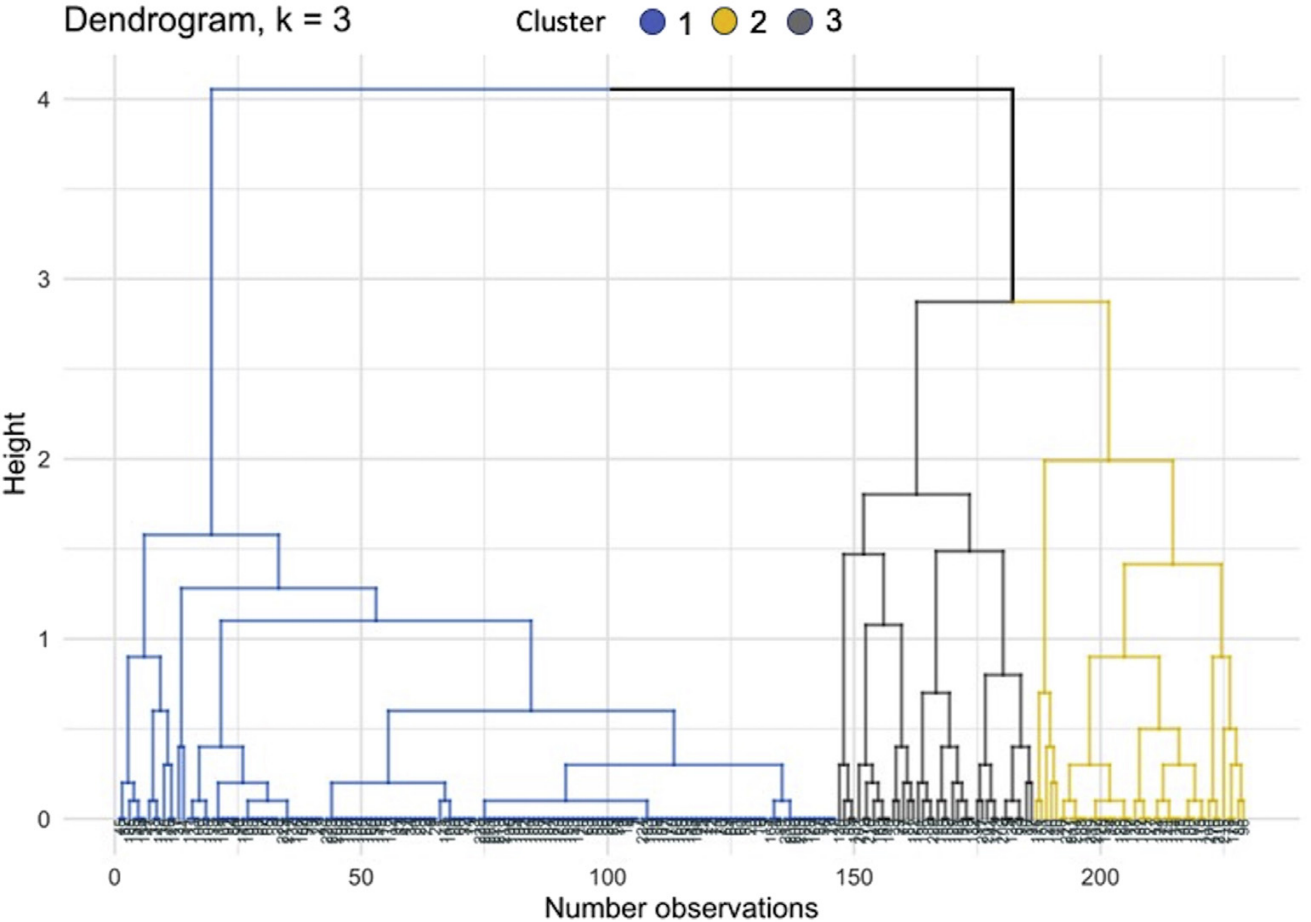
Hierarchical clustering of 229 *S. aureus* strains recovered from bovine intramammary infections in dairy herds (n = 91) from Colombia, based on both the genotypes and measured optical density (OD). The blue branch represents cluster 1 (n = 146; OD: ≤1.1), yellow to cluster 2 (n = 40; OD: ≥2.4), and grey to cluster 3 (n = 43; OD: 1.3–2.7).

**Fig. 2:**
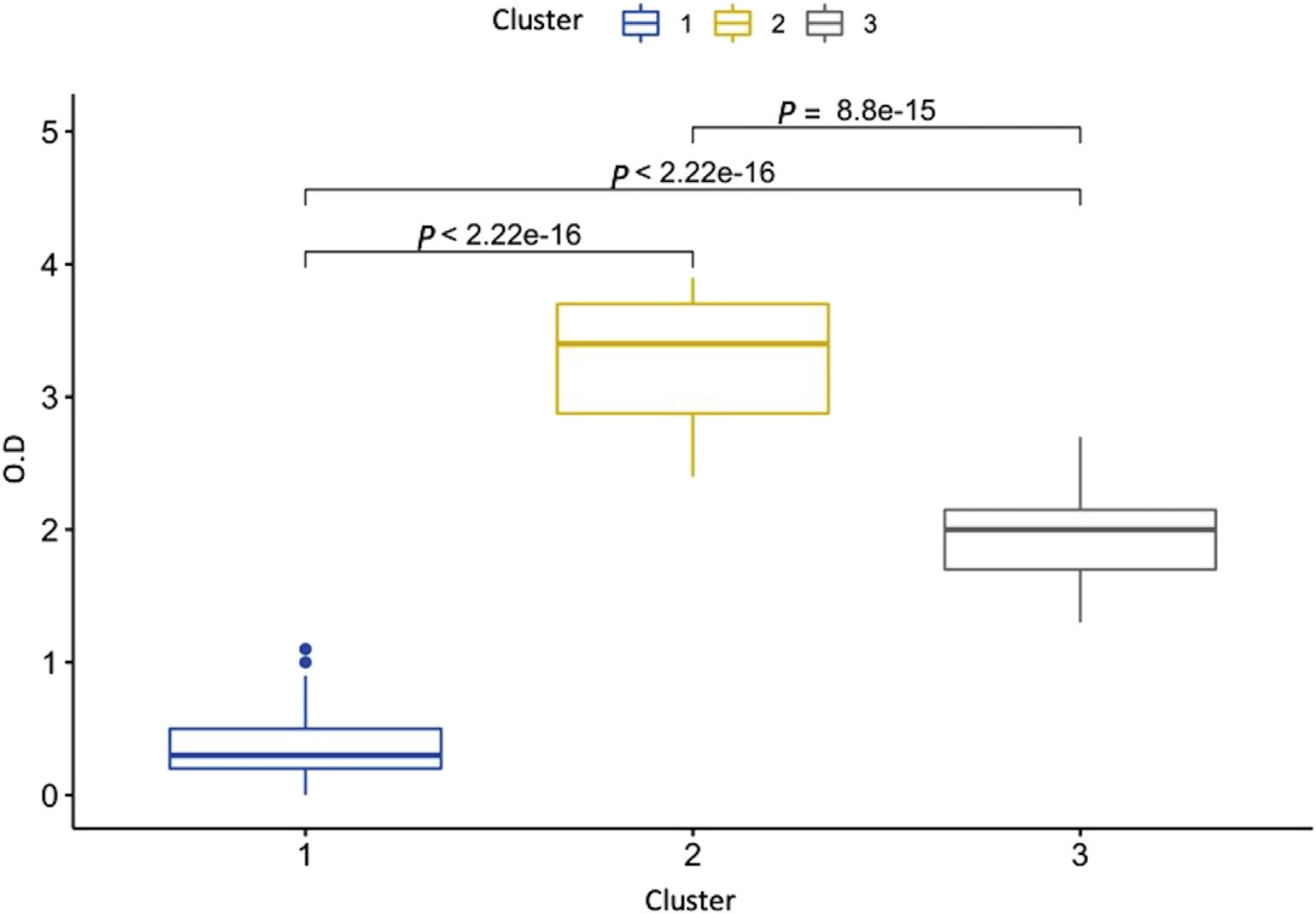
Box plot representation of distribution of the optical densities (OD) of *S. aureus* strains (n = 229) recovered from IMI in dairy herds (n = 91) from Colombia grouped by clusters (k = 3). The Kruskal-Wallis test showed significant differences among clusters (*P* < 0.0001). The blue box represents cluster 1 (n = 146; OD: ≤1.1), yellow to cluster 2 (n = 40; OD: ≥2.4), and grey to cluster 3 (n = 43; OD: 1.3–2.7).

**Fig. 3:**
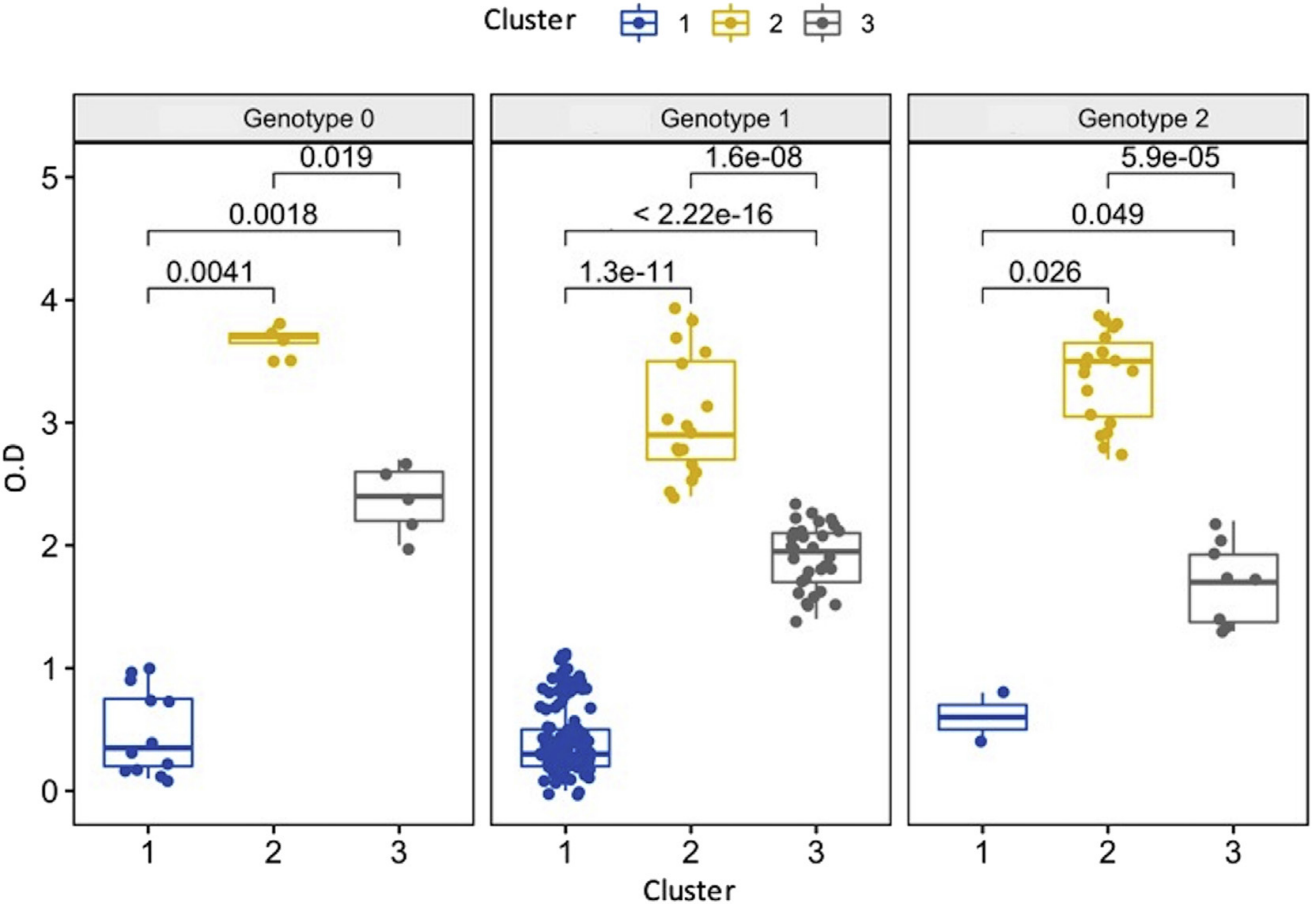
Box plot of the distribution of the optical densities (OD) of *S. aureus* strains (n = 229) recovered from IMI in Colombian dairy herds (n = 91) by each cluster (k = 3) and grouped by genotypes (n = 3). Dots represent the specific observations by group of cluster and genotypes. Significant differences were presented as Pairwise unpaired comparisons Wilcoxon test *P* values. The box and blue dots represent cluster 1 (n = 146; OD: ≤1.1), yellow to cluster 2 (n = 40; OD: ≥2.4), and grey to cluster 3 (n = 43; OD: 1.3–2.7). Most of the strains from genotype 1 (73.74%) and genotype 0 (57.14%) were classified into cluster 1, while a high percentage (65.51%) of isolates from genotype 2 were linked to cluster 2.

Three clusters were created through hierarchical clustering analysis (Fig. 1 and Table 4). The strains that presented OD values ≤1.1 were included into the first cluster (Cluster 1), while the second cluster (Cluster 2) was formed by strains that reached higher OD values (2.4–3.9). Most of the strains from genotype 1 (132/179–73.74%) and genotype 0 (12/21–57.14%) were classified into the first cluster, while a high percentage (19/29–65.51%) of isolates from genotype 2 were linked to the second cluster. The third cluster (Cluster 3) was formed by isolates exhibiting intermediate OD values (1.3–2.7). The Kruskal-Wallis analysis found significant differences among clusters (*P* < 0.0001) (Figs. 2 and 3).

**Table 4:** Distribution of the genotypes by clusters obtained using hierarchical analysis.

Cluster (OD)	Genotype			Total
	1 No. Strains (%)	2 No. Strains (%)	0 No. Strains (%)	
1 (≤1.1)	**132 (73.74)**	2 (6.90)	**12 (57.14)**	146
2 (2.4–3.9)	17 (9.50)	**19 (65.52)**	4 (19.05)	40
3 (1.3–2.7)	30 (16.76)	8 (27.59)	5 (23.81)	43
Total	179	29	21	229

Bold numbers indicate the most frequent cluster by genotype.

## 4 Discussion

The biofilm formation ability of *S. aureus* is an important virulence factor associated with chronic IMI, which can be difficult to detect within herds (Cucarella et al., 2004; Gomes et al., 2016). On a regular basis, subclinical mastitis diagnosis is performed using the California mastitis test (CMT) and somatic cell count (SCC) (Costa et al., 2018). However, Petzer et al. (2017) demonstrated that when a SCC threshold of 200.000 cells/mL in quarter milk samples or 150.000 cells/mL in composite milk samples were used to identify infections caused by *S. aureus*, 20.5% of quarters and 30.8% of cows remained undetected. Moreover, these percentages could increase to 49% for quarter milk samples and to 69% of cows, if a threshold of 500.000 cells/mL is used. This is in agreement with the results presented by Cucarella et al. (2004), who also observed that 22.6% of infected cows were incorrectly classified as uninfected with the SCC criteria (<200.000 cells/mL), when in fact these cows were infected with the genotypes best capable of forming biofilms. These microorganisms can remain undetected in the udder, the biofilm-forming strains may cause misdiagnosis of IMI, allowing the persistence of this pathogen in dairy herds (Costa et al., 2018; Cucarella et al., 2004). In this work, we found that 81.26% of *S. aureus* isolates were biofilm formers *in vitro*, which supports results shown by Coelho et al. (2011) and Marques et al. (2013) in Brazil, as well as Bardiau et al. (2014) in Belgium, 80%, 81%, and 82.5%, respectively. The percentages identified in this study, however, were lower than reported by Pereyra et al. (2016) in Argentina, Serray et al. (2016) in Morocco, Salimena et al. (2016) and Marques et al. (2017) in Brazil, and Notcovich et al. (2018) in New Zealand, where the percentage of isolates that were biofilm formers ranged between 93.4% to 100%. The causes of this discrepancy may be due to genotypic characteristics of the strains from each geographical region, or could also be due to the differences in methodologies used to determine biofilm-forming ability (Aslantaş and Demir, 2016).

*S. aureus* clinical isolates have been described by diferent reports as carriers of the *ica* operon (Cucarella et al., 2004; McCarthy et al., 2015). This operon is formed by four genes which are necessary for synthesis of PIA, one of the main components of the biofilm matrix which promotes intercellular aggregation (McCarthy et al., 2015). Unlike most studies, where a high prevalence (generally 100%) of *ica* genes has been reported (Szweda et al., 2012; Pereyra et al., 2016; Salimena et al., 2016; Serray et al., 2016; Marques et al., 2017), we identified this operon in 90.82% of the strains, which agrees with results from studies conducted in Turkey (86.6%) by Aslantaş and Demir (2016) and Spain (94.0%) by Cucarella et al. (2004). Some authors have suggested that failure in the amplifications of *ica* gene with PCR could occur due to the absence of the operon, mutations in the target sequence of the primers or the insertion of mobile genetic elements in the operon, both of which can affect the operon integrity (Götz, 2002).

When biofilm formation was tested on polystyrene microtiter plates, 141 (78.77%) of the isolates that only harbored *ica* operon (genotype 1) were able to produce biofilms. However, most of these strains, 132 (73.74%), were linked to Cluster 1 from the hierarchical analysis. According to Cucarella et al. (2004), the strains with the presence of *ica* gene were less capable of biofilm formation (less biomass) than those carrying both *ica* and *bap* genes. This could suggest that other genes or stimuli could also play an important role in this process.

Regarding the *bap* gene, it is known that it encodes for Bap, which is an anchored surface protein implicated in PIA-independent biofilms, promoting both primary attachment and intercellular adhesion (Cucarella et al., 2004; McCarthy et al., 2015). In our study, 12.66% of the strains tested were found to harbor the *bap* gene. These results are similar to those obtained in Brazil (15.8%) by Zuniga et al. (2015), in Turkey (13.4%) by Aslantaş and Demir (2016), and Argentina (11.0%) by Felipe et al. (2017). On the other hand, Szweda et al. (2012), de Almeida et al. (2013), Serray et al. (2016), Pereyra et al. (2016) and Notcovich et al. (2018) did not detect this gene among the strains found in their respective studies. Although this gene is located in a mobile genetic element, the low frequency of detection shows that its transfer is not common (Snel et al., 2015). All of the *bap*-positive strains (genotype 2) were found to be biofilm forming, and most of them (65.52%) were included in Cluster 2 from the hierarchical analysis. Similarly, Cucarella et al. (2004) reported that the strains carrying *ica* and *bap* genes were strong biofilm producers, explaining the possible role of Bap in their higher capacity to form biofilms. Additionally, evidence has shown that this high capacity biofilm-forming genotype is more capable of persisting in the bovine mammary gland, and as such, it can be more difficult to eradicate (Cucarella et al., 2004).

PCR analysis showed that in 9.17% (21/229) of the strains tested, the *ica* and *bap* genes were not detected (genotype 0). In other studies carried out by Cucarella et al. (2004), de Almeida et al. (2013), and Aslantaş and Demir (2016), strains negative for both genes were also reported. Although these genes were not amplified in our isolates, most of the isolates 76.19% (16/21) were still capable of producing biofilms. Similar to observations with genotype 1, over half of the strains that formed biofilms were part of Cluster 1. Different authors have also reported discrepancies between the phenotypic and genotypic results obtained in biofilm assays. This situation could be explained by the diversity and redundancy of the biofilm-forming mechanisms used by *S. aureus*, a process which seems not to be solely dependent on *ica* and *bap* genes. Other reported mechanisms that play an important role in the biofilm formation process by *S. aureus* are the release of extracellular DNA (eDNA), fibrin–dependent agglomeration and accumulation mediated by amyloid aggregates (Zapotoczna et al., 2016).

## 5 Conclusions

In conclusion, the results of this study show that most of *S. aureus* strains isolated from bovine IMI in different regions are capable of developing biofilms, indicating the pathogenic potential of these strains, especially of the *bap* gene carrying strains. Likewise, these results confirm what have been previously reported; that *ica* and *bap* genes play an important role in the process of biofilm formation, but that they are not the only mechanisms involved in this process. The inclusion of knocked out strains with the abscence of *bap* and *ica* genes, will produce more conclusive results for the biofilm formation mechanism for this particular pathogen in future studies.

Our findings will contribute to the knowledge of the epidemiology of *S. aureus* in the region, and could potentially inform the application of control and management protocols for dealing with infections within herds.

## Declarations

### Author contribution statement

G. Torres: Conceived and designed the experiments; Performed the experiments; Analyzed and interpreted the data; Contributed reagents, materials, analysis tools or data; Wrote the paper.

K. Vargas: Performed the experiments.

M. Sánchez-Jiménez: Conceived and designed the experiments; Analyzed and interpreted the data.

J. Reyes-Velez: Conceived and designed the experiments; Analyzed and interpreted the data; Wrote the paper.

M. Olivera-Angel: Analyzed and interpreted the data; Contributed reagents, materials, analysis tools or data; Wrote the paper.

### Funding statement

This study was supported by Colciencias (grant number 727, 2015), University of Antioquia (grant number 2017-15551) and Biogenesis Research Group (grant number ES84180138) from Colombia.

### Competing interest statement

The authors declare no conflict of interest.

### Additional information

No additional information is available for this paper.

## References

[bib1] Aslantaş Ö., Demir C. (2016). Investigation of the antibiotic resistance and biofilm-forming ability of *Staphylococcus aureus* from subclinical bovine mastitis cases. J. Dairy Sci..

[bib2] Bardiau M., Detilleux J., Farnir F., Mainil J.G., Ote I. (2014). Associations between properties linked with persistence in a collection of *Staphylococcus aureus* isolates from bovine mastitis. Vet. Microbiol..

[bib3] Brakstad O.G., Aasbakk K., Maeland J.A. (1992). Detection of *Staphylococcus aureus* by polymerase chain reaction amplification of the nuc gene. J. Clin. Microbiol..

[bib4] Coelho S.M.O., Pereira I.A., Soares L.C., Pribul B.R., Souza M.M.S. (2011). Short communication: profile of virulence factors of *Staphylococcus aureus* isolated from subclinical bovine mastitis in the state of Rio de Janeiro, Brazil. J. Dairy Sci..

[bib5] Costa F.N., Belo N.O., Costa E.A., Andrade G.I., Pereira L.S., Carvalho I.A., Santos R.L. (2018). Frequency of enterotoxins, toxic shock syndrome toxin-1, and biofilm formation genes in *Staphylococcus aureus* isolates from cows with mastitis in the Northeast of Brazil. Trop. Anim. Health Prod..

[bib6] Cucarella C., Tormo M.A., Ubeda C., Trotonda M.P., Monzón M., Peris C., Penadés J.R. (2004). Role of biofilm-associated protein bap in the pathogenesis of bovine *Staphylococcus aureus*. Infect. Immun..

[bib7] de Almeida L.M., de Almeida M.Z., de Mendonça C.L., Mamizuka E.M. (2013). Comparative analysis of agr groups and virulence genes among subclinical and clinical mastitis *Staphylococcus aureus* isolates from sheep flocks of the Northeast of Brazil. Braz. J. Microbiol..

[bib8] Felipe V., Morgante C.A., Somale P.S., Varroni F., Zingaretti M.L., Bachetti R.A., Porporatto C. (2017). Evaluation of the biofilm forming ability and its associated genes in *Staphylococcus* species isolates from bovine mastitis in Argentinean dairy farms. Microb. Pathog..

[bib9] Fournier C., Kuhnert P., Frey J., Miserez R., Kirchhofer M., Kaufmann T., Graber H.U. (2008). Bovine *Staphylococcus aureus*: association of virulence genes, genotypes and clinical outcome. Res. Vet. Sci..

[bib10] Gomes F., Saavedra M.J., Henriques M. (2016). Bovine mastitis disease/pathogenicity: evidence of the potential role of microbial biofilms. Pathogens and Disease.

[bib11] Götz F. (2002). *Staphylococcus* and biofilms. Mol. Microbiol..

[bib12] Lasa I., Penadés J.R. (2006). Bap: a family of surface proteins involved in biofilm formation. Res. Microbiol..

[bib13] Marques V., de Souza M., Mendoca E., de Alencar T., Rocha B., Coelho S., Reinoso E. (2013). Análise fenotípica e genotípica da virulência de *Staphylococcus* spp. e de sua dispersão clonal como contribuição ao estudo da mastite bovina. Pesqui. Vet. Bras..

[bib14] Marques V.F., Motta C.C., Soares B.D., Melo D.A., Coelho S.M., Coelho I.D., Souza M.M. (2017). Biofilm production and beta-lactamic resistance in Brazilian *Staphylococcus aureus* isolates from bovine mastitis. Braz. J. Microbiol..

[bib15] McCarthy H., Rudkin J.K., Black N.S., Gallagher L., O’Neill E., O’Gara J.P. (2015). Methicillin resistance and the biofilm phenotype in *Staphylococcus aureus*. Frontiers in Cellular and Infection Microbiology.

[bib16] National Mastitis Council (2004). Microbiological Procedures for the Diagnosis of Bovine Udder Infection and Determination of Milk Quality.

[bib17] Notcovich S., DeNicolo G., Flint S.H., Williamson N.B., Gedye K., Grinberg A., Lopez-Villalobos N. (2018). Biofilm-forming potential of *Staphylococcus aureus* isolated from clinical mastitis cases in New Zealand. Veterinary Sciences.

[bib18] Pereyra E.A.L., Picech F., Renna M.S., Baravalle C., Andreotti C.S., Russi R., Dallard B.E. (2016). Detection of *Staphylococcus aureus* adhesion and biofilm-producing genes and their expression during internalization in bovine mammary epithelial cells. Vet. Microbiol..

[bib19] Petzer I.-M., Karzis J., Donkin E.F., Webb E.C., Etter E.M.C. (2017). Validity of somatic cell count as indicator of pathogen-specific intramammary infections. J. S. Afr. Vet. Assoc..

[bib20] Salimena A.P.S., Lange C.C., Camussone C., Signorini M., Calvinho L.F., Brito M.A.V.P., Piccoli R.H. (2016). Genotypic and phenotypic detection of capsular polysaccharide and biofilm formation in *Staphylococcus aureus* isolated from bovine milk collected from Brazilian dairy farms. Vet. Res. Commun..

[bib21] Schukken Y., Günther J., Fitzpatrick J., Fontaine M.C., Goetze L., Holst O., Seyfert H.-M. (2011). Host-response patterns of intramammary infections in dairy cows. Vet. Immunol. Immunopathol..

[bib22] Serray B., Oufrid S., Hannaoui I., Bourjilate F., Soraa N., Mliji M., El Azhari M. (2016). Genes encoding adhesion factors and biofilm formation in methicillin-resistant *Staphylococcus aureus* in Morocco. Journal of Infection in Developing Countries.

[bib23] Snel G.G.M., Monecke S., Ehricht R., Piccinini R. (2015). Molecular characteristics of bap-positive *Staphylococcus aureus* strains from dairy cow mastitis. J. Dairy Res..

[bib24] Stepanović S., Vuković D., Hola V., Di Bonaventura G., Djukić S., Cirković I., Ruzicka F. (2007). Quantification of biofilm in microtiter plates: overview of testing conditions and practical recommendations for assessment of biofilm production by staphylococci. APMIS: Acta Pathologica, Microbiologica, et Immunologica Scandinavica.

[bib25] Szweda P., Schielmann M., Milewski S., Frankowska A., Jakubczak A. (2012). Biofilm production and presence of ica and bap genes in *Staphylococcus aureus* strains isolated from cows with mastitis in the eastern Poland. Pol. J. Microbiol..

[bib26] Vautor E., Abadie G., Pont A., Thiery R. (2008). Evaluation of the presence of the bap gene in *Staphylococcus aureus* isolates recovered from human and animals species. Vet. Microbiol..

[bib27] Veh K.A., Klein R.C., Ster C., Keefe G., Lacasse P., Scholl D., Malouin F. (2015). Genotypic and phenotypic characterization of *Staphylococcus aureus* causing persistent and nonpersistent subclinical bovine intramammary infections during lactation or the dry period. J. Dairy Sci..

[bib28] Vidal J., Vargas K., Parra L., Rivera A., Macias D., Torres G., Olivera M. (2016). Prevalence of mastitis causing bacteria isolated in two diagnostic laboratories in Antioquia (Colombia), between the years 2013 and 2015. J. Vet. Sci. Technol..

[bib29] Zapotoczna M., O’Neill E., O’Gara J.P. (2016). Untangling the diverse and redundant mechanisms of *Staphylococcus aureus* biofilm formation. PLoS Pathog..

[bib30] Zuniga E., Melville P.A., Saidenberg A.B.S., Laes M.A., Gonsales F.F., Salaberry S.R.S., Benites N.R. (2015). Occurrence of genes coding for MSCRAMM and biofilm-associated protein Bap in *Staphylococcus* spp. isolated from bovine subclinical mastitis and relationship with somatic cell counts. Microb. Pathog..

